# Meningoencephalocele causing recurrent meningitis

**DOI:** 10.1002/ccr3.1420

**Published:** 2018-03-05

**Authors:** Rogério Ruas, Natália Ribeiro

**Affiliations:** ^1^ Infectious Diseases Department Centro Hospitalar São João Alameda Professor Hernani Monteiro 4200 Porto Portugal

**Keywords:** Meningitis, meningoencephalocele, recurrent, *Streptococcus*

## Abstract

Patients with recurrent meningitis must be carefully studied to exclude risk factors including anatomic defects.

A 42‐year‐old woman presented at the emergency department with fever, chills, and headache progressing in a few hours to prostration. On admission, she had anisocoria with papillary edema bilaterally but no other changes on physical examination. Brain CT found no abnormalities, but lumbar puncture confirmed bacterial meningitis with pleocytosis, increase in protein levels, and low glucose, with culture positive for *Streptococcus pneumoniae*. She was started on Ceftriaxone and dexamethasone with great clinical improvement over the next days. She had a history of a car accident with head trauma 9 years before in Brazil, and she had been treated for bacterial meningitis 3 years before, although no medical information was available. She had been asymptomatic since then and had not received pneumococcal vaccination. A brain MRI was performed suspecting risk factors for recurrent meningitis, and it showed fracture of the cribiform plate with a meningoencephalocele extending to the right superior turbinate (Fig ;1‐ panels A, B, and C). She completed 14 days of Ceftriaxone and was discharged asymptomatic. She is scheduled for anatomical correction. This report shows the need to rule out meningoencephalocele in all patients with head trauma and recurrent meningitis (Fig. 1).

**Figure 1:**
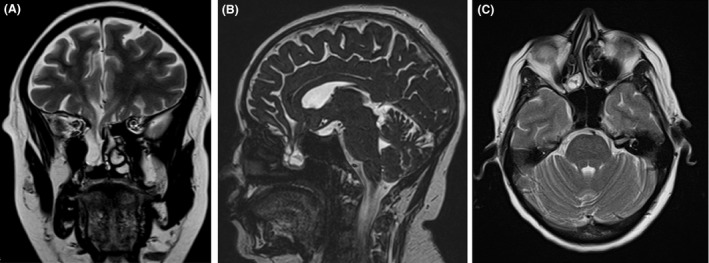
Panel A: Coronal cut: Meningoencephalocele extending to the right superior turbinate. Panel B: Sagittal cut: Meningoencephalocele extending to the right superior turbinate. Panel C: Axial cut: Meningoencephalocele extending to the right superior turbinate.

## Authorship

Both authors: managed the clinical case. Rogério Ruas: drafted the manuscript. Natália Ribeiro: revised the manuscript.

## Conflict of Interest

None declared.

